# Fitzpatrick Skin Phototype Is Independently Associated with Differential Short-Term Cutaneous Reactivity Following Standardized Topical Provocation in Humans

**DOI:** 10.3390/life16020364

**Published:** 2026-02-22

**Authors:** Laura Maghiar, Corina Beiușanu, Corina Moisa, Timea Claudia Ghitea, Octavia Gligor, Antonia Maria Lestyan, Marieta Lestyan, Ilarie Brihan, Teodor-Andrei Maghiar, Csaba Nagy, Mădălin Florin Ganea, Laura Grațiela Vicaș, Mariana Ganea

**Affiliations:** 1 Psychoneuroscience and Recovery Department, University of Oradea, 1st Decembrie Street, 410028 Oradea, Romania; laura.maghiar@uoradea.ro (L.M.); ibrihan@uoradea.ro (I.B.); 2 Department of Preclinics, Faculty of Medicine and Pharmacy, University of Oradea, 410068 Oradea, Romania; beiucorina@yahoo.com (C.B.); octavia.gligor@uoradea.ro (O.G.); antonia.lestyan@uoradea.ro (A.M.L.); marietalestyan@yahoo.com (M.L.); 3 Pharmacy Department, Faculty of Medicine and Pharmacy, University of Oradea, 1st December Square 10, 410028 Oradea, Romania; corinamoisa@hotmail.com (C.M.); lvicas@uoradea.ro (L.G.V.); mganea@uoradea.ro (M.G.); 4 Clinical County Emergency Hospital Oradea, 65 Gh. Doja Street, 410169 Oradea, Romania; teodormaghiar@yahoo.com; 5 Doctoral School of Biomedical Sciences, University of Oradea, 1 Universitătii Street, 410073 Oradea, Romania; nagycsaba95@yahoo.com; 6 Faculty of Medicine and Pharmacy, University of Oradea, 1st December Square 10, 410028 Oradea, Romania; ganea.madalinflorin@student-uoradea.ro

**Keywords:** Fitzpatrick skin phototype, cutaneous reactivity, human phenotypic variability, topical provocation, inflammatory skin response, pigmentation-related traits

## Abstract

Background: Human cutaneous reactivity exhibits marked inter-individual variability, yet the contribution of constitutional pigmentation traits to short-term skin responses remains incompletely characterized. Fitzpatrick skin phototype reflects stable differences in pigmentation-related traits and may therefore act as a phenotypic modifier of early cutaneous reactivity following topical exposure. Methods: In this controlled human study, 239 healthy volunteers were stratified by Fitzpatrick skin phototype into three groups: I–II (*n* = 138), III (*n* = 72), and IV–V (*n* = 29). A standardized emulgel-based topical provocation model was applied under occlusion to the volar forearm, and cutaneous responses were assessed at 20 min (Test A), 24 h (Test B), and 96 h (Test C) using standardized visual scoring. Group comparisons, multivariable linear regression models adjusted for age, sex, country of origin, and experimental lot, and stratified analyses by country of origin, were performed. Results: Early and short-term cutaneous responses differed significantly across phototype groups. Participants with phototypes I–II exhibited higher response scores at both 20 min and 24 h compared with phototype III (*p* < 0.001). In adjusted models, phototype III remained independently associated with significantly lower reactivity relative to phototypes I–II at 20 min (β = −1.61, *p* < 0.001) and 24 h (β = −0.98, *p* < 0.001). Responses among phototypes IV–V were minimal to absent; however, this subgroup was underrepresented, and findings for IV–V are descriptive. Age was a significant positive predictor of response intensity, whereas sex showed no independent association. No persistent reactions were observed at 96 h in any phototype group. Stratified analyses confirmed that the reduced reactivity associated with phototype III was independent of country of origin. Conclusions: Fitzpatrick skin phototype is independently associated with early and short-term cutaneous reactivity following standardized topical provocation in humans. Lighter phototypes (I–II) demonstrate increased susceptibility to transient inflammatory responses, whereas phototype III shows markedly reduced reactivity. These findings support the role of skin phototype as a constitutional modifier of short-term cutaneous responses and highlight the importance of considering pigmentation-related phenotypes in the design and interpretation of dermatological testing, cosmetic tolerability studies, and safety assessments of topical formulations.

## 1. Introduction

Melanin is the major pigment of human skin and acts as a complex biopolymer synthesized by melanocytes within specialized organelles known as melanosomes, which are subsequently transferred to keratinocytes. Two major structural forms of melanin are recognized—eumelanin and pheomelanin—and these are distributed differently across skin phenotypes. Eumelanin predominates in darker skin phenotypes and provides superior biological protection, whereas pheomelanin is relatively more abundant in lighter Fitzpatrick phototypes (I–II) and is less efficient in neutralizing free radicals [1,2,3,4]. In addition to quantitative differences, Fitzpatrick phototypes vary in melanosome architecture and distribution: in phototypes III–VI, melanosomes are larger, more numerous, and more uniformly dispersed within keratinocytes, persisting up to the stratum corneum; in contrast, melanosomes in phototypes I–II are smaller, tend to aggregate, and undergo earlier degradation [1,5]. These structural differences are associated with distinct patterns of cutaneous resilience, oxidative-stress handling, and irritant sensitivity. In addition, experimental evidence suggests that pigmentation-related traits may influence early inflammatory signaling pathways, including keratinocyte activation, cytokine release, and neurogenic inflammation, which are directly relevant to acute irritant responses.

Beyond structural pigmentation differences, accumulating evidence suggests that skin phototype may also be associated with variability in early inflammatory signaling following cutaneous exposure. Keratinocyte-derived cytokines, neurogenic inflammation, and vascular reactivity contribute to visible erythema and irritation, and inter-individual variability in these pathways has been documented in irritant and sensitive skin models. While these responses are multifactorial, pigmentation-related traits may interact with these pathways by modulating oxidative balance and local inflammatory thresholds. Importantly, most available data derive from UV-related or disease-oriented models, whereas controlled comparisons in standardized topical provocation settings remain limited [6,7].

Beyond its role in pigmentation, melanin has been proposed to influence cutaneous homeostasis through multiple pathways relevant to topical exposure. In particular, prior work supports an antioxidant role for melanin, including the capacity to interact with reactive oxygen and nitrogen species (ROS/RNS), which may contribute to attenuated inflammatory activation and improved functional resilience in more pigmented skin [2,5,6]. In the context of exposure to bioactive or chemically reactive topical compounds, these mechanisms could plausibly contribute to phototype-dependent differences in erythema and irritation thresholds. However, the present study did not directly quantify oxidative stress or inflammatory mediators; therefore, mechanistic interpretations are presented as hypothesis-driven context rather than demonstrated causal pathways [2,5,8].

The relationship between skin phototype and epidermal barrier function is complex and not uniformly defined. Some studies report greater corneocyte cohesion and distinct barrier recovery kinetics in more pigmented skin, whereas others find minimal baseline differences in transepidermal water loss under resting conditions. These mixed findings suggest that phototype-related differences may become more evident under stress or irritant exposure rather than under basal conditions. This distinction is particularly relevant when interpreting provocation tests designed to elicit short-term inflammatory responses [9].

A further hypothesis supported by prior literature is that pigmentation-related traits may influence intradermal compound distribution, although this remains speculative in human in vivo settings [10,11,12,13]. If relevant for the constituents of oregano essential oil (e.g., phenolic monoterpenes), such binding could hypothetically contribute to differences in compound distribution. However, this remains a theoretical consideration in the absence of direct measurements and should not be interpreted as a demonstrated mechanism in the present study. Because we did not measure intradermal concentrations or compound–melanin interactions, we report this as a theoretical framework rather than a conclusion [1,5].

Clinical research on sensitive skin further supports the presence of phototype-associated variability in irritant perception and reactivity. Lactic acid sting tests and questionnaire-based assessments repeatedly show higher reactivity in lighter phototypes, although subjective perception and objective erythema do not always correlate. These discrepancies highlight the need for standardized experimental models that allow cross-phototype comparisons under controlled exposure conditions [14].

Structural differences in the epidermal barrier further complement these phototype-associated effects. Darker skin phototypes have been associated in some studies with greater corneocyte cohesion and distinct barrier recovery kinetics. However, findings under basal conditions are mixed, and differences may relate more to corneocyte organization and barrier repair dynamics than to uniformly stronger baseline barrier function [15]. At a functional level, clinical and experimental studies on “sensitive skin” consistently demonstrate that lighter phototypes (I–II) are more prone to erythema, stinging, and burning sensations. Provocation tests, such as the lactic acid stinging test, repeatedly show a lower irritancy threshold in phototypes I–II compared with the minimal reactivity commonly observed in phototypes III–VI [14,16,17]. These differences are not merely sensory in nature but are consistent with underlying biological variation in pigmentation-related traits, epidermal architecture, and neurogenic inflammatory responses.

Despite increasing recognition of phototype-dependent differences in skin biology, comparative clinical data evaluating short-term cutaneous responses to bioactive topical compounds across Fitzpatrick phototypes remain limited, particularly in controlled experimental settings. Moreover, many dermatological and cosmetic tolerance studies are still disproportionately conducted in lighter skin phototypes, limiting the generalizability of safety and tolerability assessments.

The aim of this study was to evaluate early and short-term cutaneous responses to a bioactive topical formulation across different Fitzpatrick skin phototypes, with a specific focus on comparing phototypes I–II, III, and IV–V. By integrating clinical response data with stratified and adjusted analyses accounting for age, sex, and country of origin, this work seeks to clarify the extent to which skin phototype independently modulates cutaneous reactivity, thereby contributing to more inclusive and biologically informed human cutaneous response research.

## 2. Materials and Methods

### 2.1. Standardized Topical Provocation Model

A standardized emulgel-based topical provocation model was prepared for controlled human cutaneous response testing. The formulation was developed exclusively for experimental use and was not intended as a marketed cosmetic or therapeutic product.

The provocation model consisted of an emulgel matrix incorporating a low-dose bioactive component (0.5% *w*/*w* essential oil derived from *Origanum vulgare* L.), selected to provide a reproducible, mild chemical stimulus suitable for short-term occlusive patch testing. The choice of concentration was guided by prior pilot testing and existing literature indicating low irritancy potential under controlled exposure conditions.

The aqueous phase was prepared by dispersing xanthan gum (2% *w*/*w*) and propanediol (3% *w*/*w*) in purified water under continuous stirring. The oil phase consisted of hydrogenated olive oil unsaponifiables (5% *w*/*w*), apricot kernel oil (7% *w*/*w*), ceramides (2% *w*/*w*), and a skin-conditioning peptide (1% *w*/*w*), combined under controlled heating. The two phases were homogenized using a rotor–stator system to ensure uniform dispersion of the bioactive component and consistent texture across batches. A phenoxyethanol/ethylhexylglycerin-based preservative system (1% *w*/*w*) was included to ensure microbiological stability during the testing period.

The formulation was prepared in multiple experimental lots to support reproducibility and to allow adjustment for lot-to-lot variability in the statistical analysis. Organoleptic properties and pH were assessed to confirm batch consistency prior to use. Each experimental lot included approximately 34 participants (*n* = 204 in lot-based testing), while the full analytical dataset comprised 239 participants.

All cosmetic-grade raw materials were obtained from certified European suppliers commonly used in dermatological formulation research (details provided in Supplementary Materials). The physicochemical and antimicrobial evaluations were conducted to ensure formulation consistency and safety, not as primary study outcomes. These details are reported in Supplementary Materials.

### 2.2. Supplementary Physicochemical and Antimicrobial Characterization

To ensure consistency and reproducibility of the topical provocation model, additional physicochemical and antimicrobial assessments were performed prior to in vivo testing. These included compositional verification of the bioactive component, stress-based physical stability testing, and in vitro antimicrobial assays conducted according to standardized laboratory protocols.

The essential oil of *Origanum vulgare* L. from Sigma-Aldrich^®^ (St. Louis, MO, USA) (concentration: 40 µg per 6 mm disc equivalent) was sourced from a commercial supplier specializing in pharmaceutical- and cosmetic-grade botanical extracts, with standardized composition and documented quality control.

These analyses were performed to document formulation uniformity and to exclude overt instability or contamination that could confound the interpretation of cutaneous response data. Supplementary Materials provide detailed formulation composition and quality-control parameters relevant to reproducibility (Supplementary Table S1).

### 2.3. In Vivo Assessment of Cutaneous Irritability

In vivo skin tolerability testing was performed in an authorized dermatology clinic following written informed consent from all participants. Only individuals with clinically healthy skin at the test site and without visible lesions or active dermatoses were included.

Occlusive patch testing was carried out by applying 100 µL of OvO emulgel to the volar forearm using Finn Chamber^®^ (SmartPractice, Phoenix, AZ, USA) aluminum discs (8 mm diameter; 0.5 cm^2^ area) mounted on hypoallergenic adhesive tape. Dose density was standardized to 200 µL/cm^2^ using a calibrated micropipette (AHN Biotechnologie, Nordhausen, Germany). A control patch containing the base emulgel (OvOF) and a blank patch were applied in parallel to account for vehicle and mechanical effects.

Patches were maintained under controlled environmental conditions (22 ± 1 °C; 45 ± 5% relative humidity) and removed after 24 h. Cutaneous responses—defined as erythema, irritation, and inflammatory signs—were evaluated at 20 min (Test A), 24 h (Test B), and 96 h (Test C) following application.

**Figure 1:**
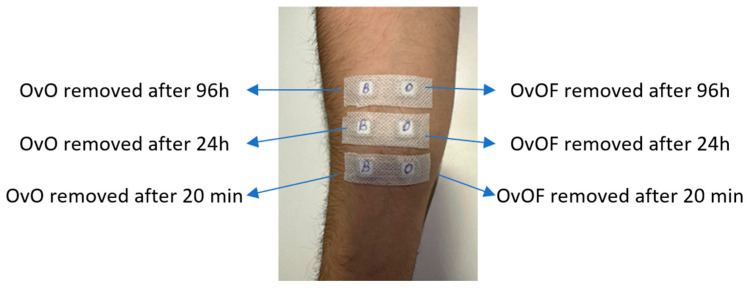
Experimental design for application and removal of patches used for in vivo testing of possible irritant reactions of OvOF and OvO emulgel.

Participants were grouped according to sex, age, and Fitzpatrick skin phototype, allowing for comparative analysis of phototype-dependent cutaneous responses (Figure 1).

#### Fitzpatrick Phototype Assignment

Fitzpatrick phototype was assigned at baseline by a trained clinician using a standardized Fitzpatrick questionnaire (self-reported tendency to burn and tan with first seasonal sun exposure), complemented by clinical inspection under standardized indoor lighting. To reduce misclassification related to transient tanning, participants were asked about recent sun exposure and the use of tanning beds. Individuals with marked recent tanning or sunburn at the test site were excluded or rescheduled. Phototype was treated as a baseline categorical variable for the current analysis; within-person changes in pigmentation over time were not assessed.

### 2.4. Statistical Analysis

Statistical analyses were performed using SPSS Statistics v30 (IBM Corp., Armonk, NY, USA).

Because cutaneous response scores were ordinal and not normally distributed, non-parametric methods were used for between-group comparisons. Kruskal–Wallis tests followed by Bonferroni-adjusted pairwise Mann–Whitney tests were applied.

Associations between continuous variables were explored using Pearson correlation coefficients.

Multivariable linear regression models with heteroskedasticity-consistent (HC3) robust standard errors were used to evaluate independent associations while adjusting for age, sex, country of origin, and experimental lot.

All tests were two-tailed, and *p* < 0.05 was considered statistically significant.

### 2.5. Ethical Approval

The in vivo study was approved by the Ethics Committee of the University of Oradea (approval no. CEFMF/5 din 28 March 2024), in accordance with the Declaration of Helsinki (2013 revision). Participants were enrolled in multiple experimental lots of approximately 34 individuals each; the full analytical dataset comprised 239 participants. Inclusion criteria: healthy volunteers aged 18–70 years, no active dermatoses; exclusion criteria: chronic skin disease, recent topical/systemic antibiotic or corticosteroid use.

## 3. Results

### 3.1. Study Population and Fitzpatrick Group Stratification

A total of 239 participants were included and stratified into three groups according to Fitzpatrick skin phototype: Group 1 (I–II, *n* = 138), Group 2 (III, *n* = 72), and Group 3 (IV–V, *n* = 29). Overall, sex distribution did not differ significantly across groups (χ^2^ = 2.25, *p* = 0.325). Mean age, however, differed significantly (Kruskal–Wallis H = 21.75, *p* = 1.89 × 10^−5^), driven by the substantially younger age in Group IV–V compared to both I–II and III (pairwise Mann–Whitney with Bonferroni correction: *p* = 3.40 × 10^−5^ and *p* = 3.10 × 10^−5^, respectively), while no age difference was observed between I–II and III (*p* = 1.00).

Regarding origin, the dataset was predominantly Romanian (205/239). Group-level distributions were: Romania 97.8% (I–II), 95.8% (III), and 3.4% (IV–V), indicating that the IV–V subgroup consisted mainly of non-Romanian participants.

Cutaneous response at 20 min, 24 h, and 96 h

Mean response scores (mean ± SD) are summarized below.

- Test A (20 min): Group I–II showed higher early erythema/irritation scores (1.82 ± 2.54) compared to Group III (0.14 ± 0.83) and Group IV–V (0.00 ± 0.00). Between-group differences were significant (Kruskal–Wallis H = 51.91, *p* = 5.34 × 10^−12^). Pairwise comparisons confirmed significantly greater reactivity in I–II vs. III (p_Bonf = 3.62 × 10^−9^) and I–II vs. IV–V (p_Bonf = 3.48 × 10^−5^), whereas III vs. IV–V did not differ (p_Bonf = 1.00).
- Test B (24 h): Delayed responses remained higher in Group I–II (0.99 ± 1.99) than in Group III (0.00 ± 0.00) and Group IV–V (0.03 ± 0.19), with significant overall differences (Kruskal–Wallis H = 20.86, *p* = 2.96 × 10^−5^). Pairwise testing showed I–II > III (p_Bonf = 1.28 × 10^−4^). The I–II vs. IV–V contrast did not remain significant after correction (p_Bonf = 0.073). Notably, Group III displayed no measurable 24 h response (Test B = 0 for all participants), indicating an absence of delayed reactivity within this subgroup in the current dataset.
- Test C (96 h): Scores were near-zero across all groups (I–II: 0.007 ± 0.085; III: 0.000 ± 0.000; IV–V: 0.069 ± 0.371) and did not differ significantly (Kruskal–Wallis H = 3.03, *p* = 0.220), consistent with resolution of skin reactions by 96 h in most participants.

Across timepoints, lighter phototypes (I–II) exhibited consistently higher early and short-term cutaneous responses, while phototype III and especially IV–V showed minimal to absent reactivity, particularly at 20 min.

### 3.2. Multivariable Regression Analysis of Cutaneous Responses

To account for potential confounding effects of demographic and contextual variables, multivariable linear regression analyses were performed for early (Test A, 20 min) and delayed (Test B, 24 h) cutaneous responses. All models included Fitzpatrick phototype group (I–II, III, IV–V) as the primary independent variable and were adjusted for age, sex, country of origin (Romania vs. other), and experimental lot (categorical fixed effects). Robust standard errors (HC3) were applied.

#### 3.2.1. Regression Model for Test A (20 min)

In the adjusted model for Test A (20 min), Fitzpatrick phototype remained a significant independent predictor of early cutaneous reactivity (Table 1). Compared with phototypes I–II (reference category), phototype III was associated with a significantly lower Test A score (β = −1.61, 95% CI: −2.07 to −1.15; *p* = 5.4 × 10^−12^). Clinically, this corresponds to an average reduction of approximately 1.6 points in early visual irritation/erythema scoring, shifting responses from the mild–moderate range observed in phototypes I–II toward near-absent reactivity in phototype III within this dataset. The difference between phototypes IV–V and I–II was not statistically significant after adjustment (β = −0.34, 95% CI: −1.30 to 0.62; *p* = 0.489).

Age was independently associated with increased early skin reactivity (β = +0.065 per year, 95% CI: 0.027 to 0.103; *p* < 0.001), corresponding to an approximate increase of 0.65 points per decade. Sex did not significantly influence Test A scores (*p* = 0.720). Participants originating from countries other than Romania exhibited significantly lower Test A values compared to Romanian participants (β = −0.73, 95% CI: −1.34 to −0.12; *p* = 0.019).

The overall model explained approximately 25% of the variance in Test A responses (R^2^ = 0.248; adjusted R^2^ = 0.215).

Model 1: Test A (20 min)

#### 3.2.2. Regression Model for Test B (24 h)

For delayed cutaneous responses at 24 h (Test B), multivariable regression demonstrated a consistent pattern (Table 2). Phototype III was strongly associated with lower Test B scores compared with phototypes I–II (β = −0.98, 95% CI: −1.31 to −0.64; *p* = 1.5 × 10^−8^). In practical terms, this indicates an average reduction of ~1 point in delayed (24 h) visual reaction score relative to phototypes I–II under the same occlusive exposure conditions. In contrast, no significant difference was observed between phototypes IV–V and I–II after adjustment (β = −0.24, 95% CI: −0.77 to 0.29; *p* = 0.367).

Age remained a significant positive predictor of delayed reactivity (β = +0.053 per year, 95% CI: 0.025 to 0.081; *p* < 0.001), while sex and country of origin were not independently associated with Test B outcomes (*p* = 0.743 and *p* = 0.144, respectively).

The Test B model explained approximately 21% of the variance in delayed responses (R^2^ = 0.209; adjusted R^2^ = 0.174). Notably, all participants classified as Fitzpatrick phototype III exhibited Test B values of zero, indicating a complete absence of measurable delayed skin response in this subgroup.

**Figure 2:**
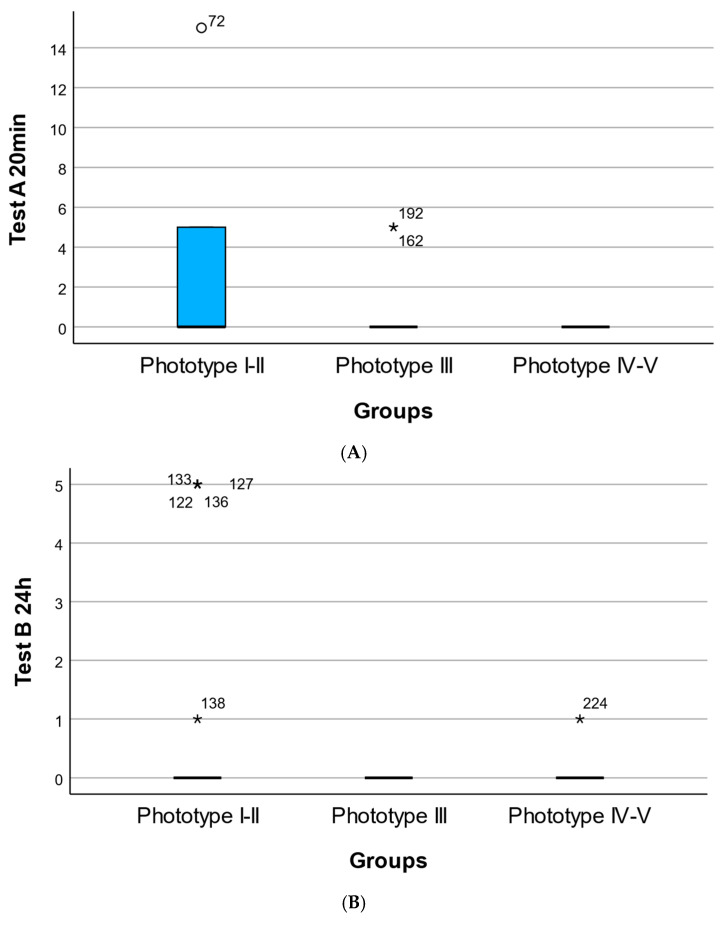

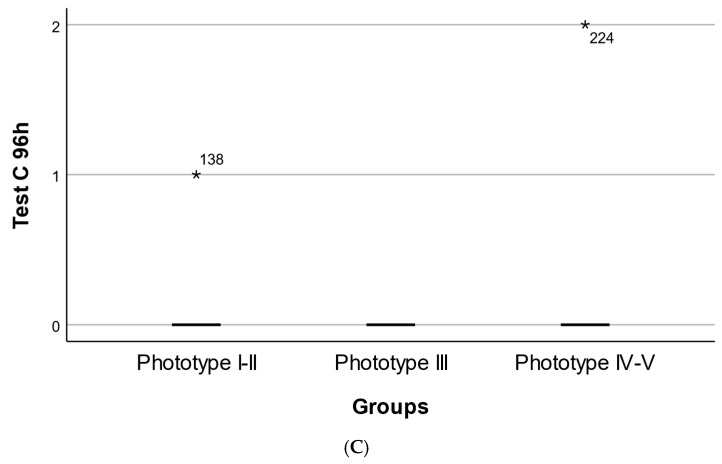
Phototype-dependent distribution of cutaneous response scores following topical application of the oregano oil emulgel. (**A**) Distribution of early cutaneous response scores at 20 min (Test A) across Fitzpatrick skin phototypes I–II, III, and IV–V. (**B**) Distribution of delayed cutaneous response scores at 24 h (Test B) across Fitzpatrick skin phototypes I–II, III, and IV–V. (**C**) Distribution of late cutaneous response scores at 96 h (Test C) across Fitzpatrick skin phototypes I–II, III, and IV–V. Each point represents an individual participant’s score. Overlaid markers indicate group summaries (median and interquartile range). Numeric labels denote sample size per group. * Represents an outlier value.

Overall, after controlling for age, sex, country of origin, and experimental lot, Fitzpatrick phototype III consistently demonstrated significantly lower early and delayed cutaneous reactivity compared with phototypes I–II, whereas phototypes IV–V did not show statistically significant differences relative to I–II in the adjusted models. Age emerged as a robust independent determinant of both early and delayed skin responses, while sex had no measurable effect. These findings support an intrinsic phototype-dependent modulation of cutaneous reactivity that is independent of major demographic confounders. Phototype-dependent distribution of cutaneous response scores following topical application of the oregano oil emulgel is presented in Figure 2.

### 3.3. Stratified Analysis by Country of Origin

Given the marked imbalance in country distribution across Fitzpatrick groups—particularly the predominance of non-Romanian participants in phototypes IV–V—a stratified analysis was performed to evaluate the robustness of phototype-related effects within more homogeneous geographic subsets. Analyses were conducted separately for Romanian participants and participants from other countries, focusing on comparisons that were statistically and biologically feasible within each stratum.

#### 3.3.1. Romanian Subgroup: Fitzpatrick I–II vs. III

Among Romanian participants (*n* = 205), only Fitzpatrick phototypes I–II (*n* = 135) and III (*n* = 69) were sufficiently represented to allow meaningful comparison; phototypes IV–V were virtually absent and therefore excluded from this stratum.

At 20 min (Test A), participants with phototypes I–II exhibited significantly higher skin response scores compared with phototype III (mean ± SD: 1.84 ± 2.56 vs. 0.15 ± 0.84, respectively). This difference was statistically significant (Mann–Whitney U test, *p* < 1 × 10^−9^).

At 24 h (Test B), delayed cutaneous responses were again higher in phototypes I–II (1.01 ± 2.02) compared with phototype III (0.00 ± 0.00), with the difference remaining highly significant (*p* < 1 × 10^−6^). Notably, all Romanian participants classified as Fitzpatrick phototype III showed complete resolution or absence of delayed response at 24 h.

At 96 h (Test C), scores were negligible in both groups, with no statistically significant differences observed.

These findings indicate that, within a geographically homogeneous Romanian population, Fitzpatrick phototype III is associated with markedly reduced early and delayed cutaneous reactivity compared with phototypes I–II, independent of country-related confounding.

#### 3.3.2. Non-Romanian Subgroup: Descriptive Comparison

The non-Romanian subgroup (*n* = 34) consisted predominantly of participants with Fitzpatrick phototypes IV–V, with only sporadic representation of phototypes I–II and III. Due to this highly unbalanced distribution, formal inferential comparisons between phototype groups were not statistically appropriate.

Descriptively, participants with phototypes IV–V in the non-Romanian subgroup exhibited minimal to absent skin responses, with Test A and Test B values approaching zero in the vast majority of cases. No persistent reactions were observed at 96 h (Test C).

Given the limited sample size and lack of internal phototype comparators within this stratum, these observations are reported descriptively and interpreted cautiously.

**Figure 3:**
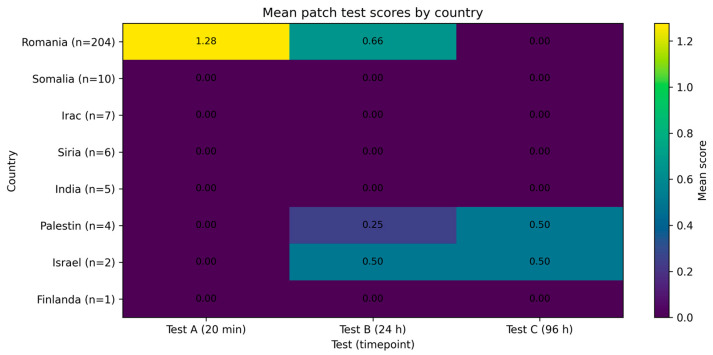
Mean cutaneous response scores at 20 min, 24 h, and 96 h stratified by country of origin. Bars represent mean values of Test A (20 min), Test B (24 h), and Test C (96 h) for each country included in the study. Differences in response magnitude across countries reflect underlying variations in participant composition, including Fitzpatrick skin phototype distribution and demographic characteristics.

Stratified analyses confirmed that the lower cutaneous reactivity observed in Fitzpatrick phototype III is not attributable to country of origin, as the effect persisted within the Romanian subgroup. In contrast, analyses involving phototypes IV–V were constrained by sample structure and were therefore limited to descriptive reporting. These results support a phototype-dependent pattern of cutaneous response that remains evident after geographic stratification (Figure 3).

### 3.4. Correlations

Pearson correlation coefficients (r) describe the relationship between Fitzpatrick skin phototype and cutaneous response scores assessed at 20 min (Test A), 24 h (Test B), and 96 h (Test C) following topical application of the oregano oil emulgel. A significant negative correlation was observed between skin phototype and both early (Test A) and delayed (Test B) responses, indicating that lighter phototypes are associated with higher cutaneous reactivity, whereas darker phototypes show reduced response intensity. No significant correlation was identified for Test C at 96 h, reflecting the absence of persistent cutaneous reactions at this time point. Statistical significance is indicated as *p* < 0.01 (Table 3).

**Figure 4:**
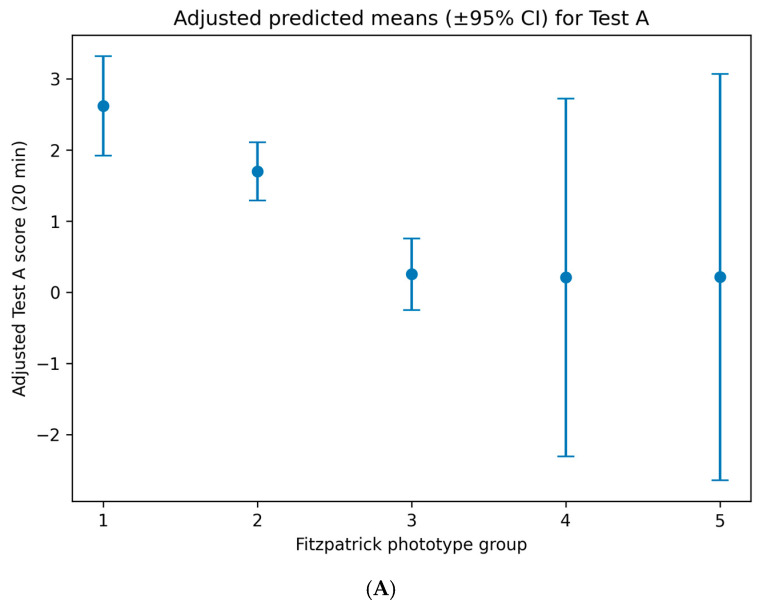

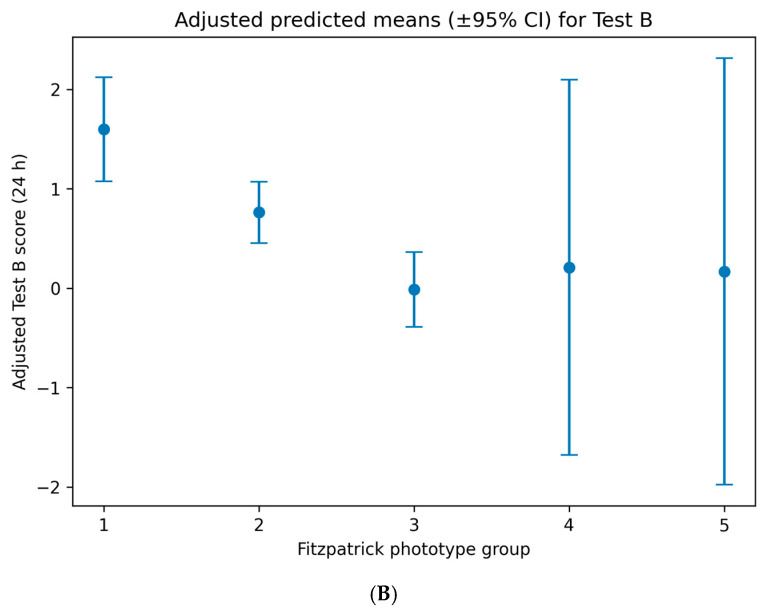
Adjusted predicted mean cutaneous response scores following topical application of the oregano oil emulgel, stratified by Fitzpatrick skin phototype. (**A**) Adjusted predicted means (±95% confidence intervals) for early response at 20 min (Test A). (**B**) Adjusted predicted means (±95% confidence intervals) for delayed response at 24 h (Test B). Fitzpatrick phototype was treated as a categorical variable in multivariable linear regression models using dummy coding, with phototypes I–II as the reference group. Predictions are adjusted for age, sex, country of origin, and experimental lot.

Each data point represents an individual participant. Regression lines indicate the direction and strength of the linear association, with corresponding regression equations and coefficients of determination (R^2^) displayed on each panel. In both models, a negative slope is observed, indicating that higher Fitzpatrick phototypes (darker skin types) are associated with lower cutaneous response scores. The association is stronger for Test A (R^2^ = 0.136) than for Test B (R^2^ = 0.086), consistent with a more pronounced phototype-dependent modulation of early cutaneous reactivity (Figure 4).

## 4. Discussion

The present study demonstrates that short-term cutaneous reactivity to a standardized topical provocation is significantly associated with Fitzpatrick skin phototype in humans, independently of age, sex, and country of origin. The magnitude of age differences across phototype groups may introduce residual confounding despite statistical adjustment. Therefore, phototype-related differences should be interpreted with caution, particularly in unadjusted comparisons. Across unadjusted analyses, multivariable regression models, and stratified geographic comparisons, lighter phototypes (I–II) consistently exhibited higher early and delayed response scores, whereas phototype III showed markedly reduced reactivity, and phototypes IV–V displayed minimal to absent responses. The findings support the interpretation that phototype is associated with variability in short-term cutaneous responses and may serve as a practical constitutional marker in this context.

### 4.1. Phototype III as a Functional Transition Phenotype

One of the most robust and reproducible findings of this study is the distinct behavior of Fitzpatrick phototype III. In both early (20 min) and delayed (24 h) assessments, phototype III exhibited near-absent responses, including a complete absence of measurable delayed reactivity at 24 h in the present dataset. Importantly, this pattern persisted after adjustment for major demographic covariates and was confirmed within a geographically homogeneous subgroup, indicating that the observed effect is not attributable to confounding by age, sex, or country of origin [18,19,20].

These results suggest that phototype III may represent a functional transition phenotype in the context of visually assessed short-term cutaneous responses. While Fitzpatrick phototypes are often grouped broadly in clinical and experimental studies, the present findings indicate that such grouping may obscure biologically meaningful differences in cutaneous responsiveness [19,21]. The identification of phototype III as a distinct response category has implications for the interpretation of human skin studies that rely on visual inflammatory endpoints and supports more refined phototype stratification in future research [22].

### 4.2. Biological Interpretation Without Causal Attribution

The observed gradient of cutaneous reactivity across phototypes is consistent with known biological differences associated with pigmentation-related traits, including variations in melanin content, melanosome distribution, epidermal architecture, and barrier properties. These traits have been linked in prior studies to differential handling of inflammatory stimuli, oxidative stress, and irritant exposure [23,24]. However, the present study did not directly assess melanin content, oxidative markers, barrier integrity, or inflammatory mediators, and therefore does not permit causal attribution to any specific biological mechanism [25,26,27,28].

Accordingly, the observed associations should be interpreted as phenotypic rather than mechanistic. Fitzpatrick phototype likely integrates multiple underlying biological characteristics that together influence cutaneous responsiveness, rather than acting through a single pathway [29,30]. The present findings therefore support the use of phototype as a practical constitutional marker of response variability, while underscoring the need for future studies incorporating objective pigmentation measurements and molecular endpoints to clarify causal mechanisms [31,32,33].

### 4.3. Influence of Age and Demographic Factors

Age emerged as a significant independent predictor of both early and delayed cutaneous responses, with increasing age associated with higher response scores. This observation is consistent with established age-related changes in skin structure and function, including altered barrier repair capacity, cumulative environmental exposure, and modulation of inflammatory responses. Importantly, adjustment for age did not attenuate the association between phototype and response intensity, indicating that phototype-related differences are not simply a surrogate for age-related variability [34,35,36].

Sex did not independently influence cutaneous responses in the present study, suggesting that, under the controlled exposure conditions employed, constitutional pigmentation traits exert a stronger influence on short-term response patterns than sex-related biological differences. Country of origin showed a modest effect in early responses; however, stratified analyses demonstrated that the reduced reactivity associated with phototype III persisted within a single-country subgroup, supporting the intrinsic nature of the phototype effect [37].

### 4.4. Methodological Implications for Human Cutaneous Response Studies

An important methodological consideration highlighted by the present findings is the limitation of visual erythema-based scoring systems across diverse skin phototypes. Reliance on visual scoring alone may underestimate subclinical responses in darker phototypes. The minimal observable responses in darker skin may reflect true biological differences, reduced visual detectability of erythema in melanin-rich skin, or a combination of both [38,39]

An additional methodological challenge in cross-phototype research is the reliance on visually assessed erythema. Melanin can partially mask vascular redness, potentially reducing the visibility of inflammation in darker skin. Consequently, visually similar scores across phototypes may not always reflect equivalent biological responses. Acknowledging this limitation is essential when interpreting phototype-dependent differences in studies relying on visual scoring systems.

These observations support the integration of objective, phototype-independent assessment tools—such as colorimetry, spectrophotometry, transepidermal water loss measurements, or imaging-based techniques—in future studies. Such approaches would enhance sensitivity, improve comparability across skin types, and strengthen the biological interpretation of cutaneous response data.

### 4.5. Strengths, Limitations, and Future Directions

The strengths of this study include a relatively large human cohort, standardized exposure conditions, multiple assessment time points, and the use of multivariable and stratified analytical approaches to address potential confounding. The consistent identification of phototype III as a low-reactivity group across analytical methods strengthens the internal validity of the findings.

Several limitations should be acknowledged. First, the uneven distribution of phototypes IV–V limited inferential analyses in this subgroup; findings for IV–V are therefore descriptive and should be interpreted cautiously. Second, the absence of objective instrumental measurements limits mechanistic inference and may underestimate responses in darker skin. Third, pigmentation was classified using Fitzpatrick phototype rather than quantitative pigment assessment, which may introduce some degree of misclassification.

Future studies should aim to incorporate objective pigmentation metrics, phototype-balanced cohorts, and molecular or biophysical endpoints to clarify causal pathways underlying phototype-associated response variability. Longitudinal and repeated-exposure designs may further elucidate the stability and clinical relevance of these response patterns [40,41].

### 4.6. Overall Interpretation

Taken together, the present findings indicate that Fitzpatrick skin phototype is a meaningful constitutional modifier of early and short-term cutaneous responses in humans. Rather than reflecting solely pigmentation or UV sensitivity, phototype appears to capture broader biological variability relevant to inflammatory responsiveness and tissue reactivity. Recognition of this variability is essential for the design, interpretation, and generalizability of human cutaneous response studies.

## 5. Conclusions

This study demonstrates that Fitzpatrick skin phototype is independently associated with early and short-term cutaneous response patterns following standardized topical provocation in humans. Lighter phototypes (I–II) consistently exhibited higher early and delayed visual response scores, whereas phototype III showed markedly reduced reactivity. Phototypes IV–V displayed minimal to absent responses; however, this subgroup was underrepresented, and findings for IV–V should be interpreted as descriptive within the limits of descriptive analysis. These associations persisted after adjustment for age, sex, country of origin, and experimental lot, supporting skin phototype as a constitutional phenotypic factor associated with short-term cutaneous responsiveness.

Importantly, the present findings do not permit causal attribution to melanin or any specific biological mechanism. Fitzpatrick phototype likely reflects the integration of multiple pigmentation-related and structural skin traits that together influence inflammatory expression and tissue reactivity. The identification of phototype III as a functional transition phenotype between higher- and lower-reactivity skin highlights the limitations of broad phototype grouping and underscores the value of refined stratification in human skin research.

From a methodological perspective, these results emphasize the importance of accounting for skin phototype when designing, analyzing, and interpreting studies that rely on visual inflammatory endpoints. Failure to do so may contribute to systematic bias and reduced comparability across diverse human populations. Future investigations incorporating objective pigmentation metrics, phototype-balanced cohorts, and phototype-independent assessment tools are warranted to clarify causal pathways and enhance biological resolution.

Overall, this work supports the integration of Fitzpatrick skin phototype as a core phenotypic variable in studies of human cutaneous responses, contributing to a more nuanced and biologically informed understanding of inter-individual variability in skin reactivity.

## Figures and Tables

**Table 1:** Multivariable linear regression analysis of early cutaneous response (Test A, 20 min) according to Fitzpatrick skin phototype and demographic factors.

Predictor	β (Mean Difference)	95% CI	*p*
Fitz III vs. I–II	−1.610	[−2.068; −1.152]	5.4 × 10^−12^
Fitz IV–V vs. I–II	−0.338	[−1.296; 0.620]	0.489
Age (per 1 year)	+0.065	[0.027; 0.103]	0.000765
Male vs. Female	+0.064	[−0.285; 0.413]	0.72
Country Other vs. Romania	−0.729	[−1.338; −0.121]	0.0187

β denotes the adjusted regression coefficient (mean difference); CI, confidence interval; *p*, two-tailed *p*-value. Fitzpatrick skin phototype I–II, female sex, and Romania were used as reference categories. Age was modeled as a continuous variable (per one-year increase).

**Table 2:** Multivariable linear regression analysis of delayed cutaneous response (Test B, 24 h) according to Fitzpatrick skin phototype and demographic factors.

Predictor	β (Mean Difference)	95% CI	*p*
Fitz III vs. I–II	−0.976	[−1.314; −0.638]	1.51 × 10^−8^
Fitz IV–V vs. I–II	−0.243	[−0.772; 0.286]	0.367
Age (per 1 year)	+0.053	[0.025; 0.081]	0.000197
Male vs. Female	+0.039	[−0.192; 0.269]	0.743
Country Other vs. Romania	−0.256	[−0.600; 0.087]	0.144

β denotes the adjusted regression coefficient (mean difference); CI, confidence interval; *p*, two-tailed *p*-value. Fitzpatrick skin phototype I–II, female sex, and Romania were used as reference categories.

**Table 3:** Correlation between Fitzpatrick skin phototype and time-dependent cutaneous response scores.

**Correlations**		**Skin Phototype Fitzpatrick**
Test A 20 min	Correlation Coefficient	−0.475 **
	Sig. (2-tailed)	<0.001
	N	239
Test B 24 h	Correlation Coefficient	−0.309 **
	Sig. (2-tailed)	<0.001
	N	239
Test C 96 h	Correlation Coefficient	0.045
	Sig. (2-tailed)	0.485
	N	239

** Correlation is significant at the 0.01 level (2-tailed).

## Data Availability

All the data processed in this article are part of the research for a doctoral thesis, which is being archived in the esthetic medical office, where the interventions were performed. Data are available from the corresponding author upon reasonable request.

## Supplementary Materials

The following supporting information can be downloaded at: https://www.mdpi.com/article/10.3390/life16020364/s1, Table S1: Qualitative and quantitative composition of the base emulgel (OvOF) and oregano oil emulgel (OvO), reported in INCI nomenclature.

## Abbreviations

The following abbreviations are used in this manuscript:

CI	Confidence interval
CFU	Colony-forming units
GC–MS	Gas chromatography–mass spectrometry
MIC	Minimum inhibitory concentration
MBC	Minimum bactericidal concentration
OvO	Oregano oil–containing emulgel
OvOF	Oregano oil–free (base) emulgel
ROS	Reactive oxygen species
RNS	Reactive nitrogen species
SD	Standard deviation
SI	Stability index
TEWL	Transepidermal water loss
