# Supplementary material for: Fitzpatrick Skin Phototype Is Independently Associated with Differential Short-Term Cutaneous Reactivity Following Standardized Topical Provocation in Humans
Source: Life (Basel). 2026 Feb 22;16(2):364. doi: 10.3390/life16020364 (PMC12941670; doi:10.3390/life16020364)
Supplement: Supplementary file 1 [file life-16-00364-s001.zip › life-4156128-supplementary.pdf]

Table S1. Qualitative and quantitative composition of the base emulgel (OvOF) and oregano oil emulgel (OvO), reported in INCI nomenclature.

Ingredient (INCI Name)	Function	OvOF (% w/w)	OvO (% w/w)	Supplier/Grade
Aqua (Water)	Solvent	q.s. to 100	q.s. to 100	Purified water, laboratory grade
Xanthan Gum	Gelling agent/rheology modifier	2.0	2.0	Research grade polysaccharide
Propanediol	Humectant/solvent	3.0	3.0	Research grade
Hydrogenated Olive Oil Unsaponifiables*	Structuring agent/consistency enhancer	5.0	5.0	Research grade
Prunus Armeniaca (Apricot) Kernel Oil	Emollient	7.0	7.0	Research grade
Ceramide NP	Barrier-support ingredient	2.0	2.0	Research grade
Palmitoyl Tripeptide-1	Skin-conditioning peptide	1.0	1.0	Research grade
Phenoxyethanol (and) Ethylhexylglycerin	Preservative system (Fragard®)	1.0	1.0	Research grade
Origanum Vulgare Oil	Active ingredient (essential oil)	0.0	0.5	doTERRA (USA), lot no. 60204665

OvOF: oregano oil–free base emulgel; OvO: oregano oil–containing emulgel. Percentages are expressed as weight/weight (% w/w). The formulation was prepared as a research-grade emulgel for controlled tolerability testing and is not presented as a marketed cosmetic product. *INCI name corresponds to the olive-derived wax fraction; final designation should reflect supplier documentation.
